# Genome assembly of the ectoparasitoid wasp *Theocolax elegans*

**DOI:** 10.1038/s41597-023-02067-5

**Published:** 2023-03-22

**Authors:** Shan Xiao, Xinhai Ye, Shuping Wang, Yi Yang, Qi Fang, Fang Wang, Gongyin Ye

**Affiliations:** 1 State Key Laboratory of Rice Biology, Ministry of Agricultural and Rural Affairs Key Laboratory of Molecular Biology of Crop Pathogens and Insect Pests & Key Laboratory of Biology of Crop Pathogens and Insects of Zhejiang Province, Institute of Insect Sciences, Zhejiang University, Hangzhou, 310058 China; 2 Shanghai Institute for Advanced Study, Zhejiang University, Shanghai, 201203 China; 3 College of Computer Science and Technology, Zhejiang University, Hangzhou, 310058 China; 4 Technical Center of Shanghai Customs for Inspection and Quarantine of Animals,Plants and Foods, Shanghai Customs, Shanghai, 200135 China

**Keywords:** Entomology, Comparative genomics

## Abstract

The ectoparasitoid wasp *Theocolax elegans* is a cosmopolitan and generalist pteromalid parasitoid of several major storage insect pests, and can effectively suppress a host population in warehouses. However, little molecular information about this wasp is currently available. In this study, we assembled the genome of *T. elegans* using PacBio long-read sequencing, Illumina sequencing, and Hi-C methods. The genome assembly is 662.73 Mb in length with contig and scaffold N50 values of 1.15 Mb and 88.8 Mb, respectively. The genome contains 56.4% repeat sequences and 23,212 protein-coding genes were annotated. Phylogenomic analyses revealed that *T. elegans* diverged from the lineage leading to subfamily Pteromalinae (*Nasonia vitripennis* and *Pteromalus puparum*) approximately 110.5 million years ago. We identified 130 significantly expanded gene families, 34 contracted families, 248 fast-evolving genes, and 365 positively selected genes in *T. elegans*. Additionally, 260 olfactory receptors and 285 venom proteins were identified. This genome assembly provides valuable genetic bases for future investigations on evolution, molecular biology and application of *T. elegans*.

## Background & Summary

Postharvest infestation by insect pests largely affects both the quality and quantity of stored grains and related commodities^1,2^. Infesting insects not only consume grains, but also transport storage fungi and bacteria^3^. Quantitative and qualitative losses from insect pests during storage are estimated to amount to 20–30%^1^. To minimize losses, fumigation has been widely used worldwide. However, such massive application of pesticides has resulted in resistance development among pests as well as negative effects on nontarget organisms, human health and the environment^4,5^. In comparison, biological control using natural enemies presents a safer and more biorational approach. Currently, parasitoid wasps from Pteromalidae, Bethylidae, Braconidae and Ichneumonidae have been reported as natural enemies of many important stored product pests, and several species have been sensibly utilized in the control of insect pests in storehouses^6–8^. Parasitoids suppress host immunity by injecting virulence factors including venom during oviposition, and the progeny consume and eventually kill the hosts, effectively, sustainably and safely controlling the host population^9,10^.

The ectoparasitoid wasp *Theocolax elegans* (Westwood) is a cosmopolitan and generalist pteromalid parasitoid of many major storage insect pests^11^. Augmentative releases of *T. elegans* can effectively diminish more than 90% of the *Rhyzopertha dominica* population in stored wheat as well as insect fragments in wheat flour^12,13^. Compared to most parasitoid wasps whose hosts are restricted to one or more genera from the same order, *T. elegans* has a broader host spectrum, and is capable of parasitizing hosts of two orders: coleopteran beetles and lepidopteran moth^13,14^. Previous studies have suggested that variation in the olfactory receptor (OR) and venom repertoire among parasitoid wasps is associated with changing host range^15–17^. The highly dynamic evolution of OR genes, which discriminate odour molecules derived from hosts and host habitats, is crucial for parasitoids to locate different hosts^17–19^. Additionally, venom proteins, which regulate host immunity, development and metabolism, likely evolve quickly and adopt novel functions in response to different host species^15,16,20^. Although the biology of *T. elegans* has been studied, little genetic information is presently available, which constrains its desirability and application as a biocontrol agent.

Here, we report a high-quality chromosome-level genome assembly of *T. elegans* using a combination of PacBio long-read sequencing, Illumina short-read sequencing and Hi-C technology. The final assembly is 662.73 Mb in length with scaffold N50 of 88.8 Mb and 94.3% completeness, providing an excellent genomic resource for subsequent research on *T. elegans*. The genome contains 56.4% repeat sequences and 23,212 protein-coding genes were annotated. To elucidate the evolutionary position of *T. elegans*, phylogenomic analyses revealed that this wasp diverged from the lineage leading to subfamily Pteromalinae (*Nasonia vitripennis* and *Pteromalus puparum*) approximately 110.5 million years ago. To examine genes that might evolve adaptively, we detected 130 significantly expanded, 34 contracted families, 248 fast-evolving genes, and 365 positively selected genes in *T. elegans*. We also identified 260 OR and 285 venom proteins. This genome provides valuable resources and insight into the fundamental aspects of evolution, molecular biology and application of *T. elegans*.

## Methods

### Sampling and sequencing

*T. elegans* parasitoid wasps were initially collected from farm-stored wheat in the experimental farmlands of Huajiachi campus, Zhejiang University (Hangzhou, China) and were reared on larvae of the rice weevil host *Sitophilus oryza* in the laboratory for at least one year before genome sequencing. The genomic DNA of approximately 150 male yellow pupae was isolated using sodium dodecyl sulfate (SDS) and proteinase K digestion, followed by phenol‒chloroform extraction. Two libraries were constructed for genome sequencing. A short-read sequencing library with an insert size of 400 bp was prepared using a Truseq Nano DNA HT Sample Preparation Kit (Illumina, USA) and sequenced using the Illumina Hiseq X Ten platform at GrandOmics Biosciences Co., Ltd. (Wuhan, China). For the 20 kb long-read library, a PacBio SMRTbell library was constructed using SMRTbell Express Template Prep Kit 2.0 (PacBio, USA) and sequenced using Single-Molecule Real-Time (SMRT) cells with the PacBio Sequel sequencer in GrandOmics Biosciences Co., Ltd. (Wuhan, China). A total of 23.7 Gb and 64.33 Gb of clean data were generated from the Illumina paired-end and PacBio libraries, respectively.

For Hi-C sequencing, the library was constructed following the standard protocol as described by Belton *et al*. with some modifications^21^. In brief, 150 male yellow pupae were ground into pieces and cross-linked by incubating in 2% formaldehyde solution. Nuclei were isolated and digested with MboI, followed by marking with biotin-14-dCTP. The ligated DNA was sheared into fragments of 300 to 600 bp, and then blunt-end repaired and A-tailed, followed by purification through biotin–streptavidin-mediated pull down. The Hi-C library was amplified by PCR (12–14 cycles) and eventually quantified and sequenced using the Illumina HiSeq 2000 platform at Annoroad Gene Technology Co., Ltd. (Beijing, China).

For transcriptome sequencing, 3–5 d-old larvae (male and mixed-sexed), yellow pupae (male and female), 2–3 d-old adults (male and female) and venom glands from 3–5 d-old female adults of *T. elegans* were collected separately with three replicates. RNA was extracted using TRIzol^®^ Reagent (Invitrogen, USA) according to the manufacturer’s protocol. RNA-Seq libraries were prepared using TruSeq RNA Sample Prep Kit (Illumina, USA) and sequenced using the Illumina HiSeq X Ten platform at GrandOmics Biosciences Co., Ltd. (Wuhan, China).

### Genome assembly

The genome size of *T. elegans* was first inferred from a 17-mer distribution using Illumina paired-end reads^22^, and the genome size was estimated to be 752.6 Mb (Supplementary Fig. S1). For *de novo* genome assembly, PacBio long reads corrected with Falcon (v1.8.7) were assembled to generate an initial assembly by Wtdbg (v1.2.8) (https://github.com/ruanjue/wtdbg-1.2.8)^23^. The initial assembly was then error-corrected with SMRTlink (v4.0) (https://www.pacb.com/support/software-downloads/) and polished with Illumina reads using Pilon (v1.22)^24^. This approach generated a 662.63 Mb assembly, with 2,824 contigs and a contig N50 of 1.15 Mb (Table 1).

**Table 1:** Statistics of the *Theocolax elegans* genome assembly.

Genome assembly	Features
Genome size	662.73 Mb
Contig N50	1.15 Mb
Number of Contigs	2,824
Scaffold N50	88.81 Mb
Number of Scaffolds	1,900
BUSCO completeness	94.30%

The Hi-C sequencing reads were mapped to the assembled genome using bowtie2 (v2.3.2)^25^. Uniquely valid paired‐end reads were retained for downstream analysis. Valid interaction pairs were identified using HiC-Pro (v2.7.8)^26^, and the scaffolds were anchored, ordered and oriented to pseudochromosomes with LACHESIS (https://github.com/shendurelab/LACHESIS)^27^. A heatmap was drawn to illustrate the interaction of each chromosome. The Hi-C technique oriented and anchored 931 scaffolds (91.9% of the whole genome assembly) to seven chromosomes (Supplementary Fig. S2). The chromosome-level genome assembly was 662.73 Mb in length with 1,900 scaffolds and a scaffold N50 of 88.8 Mb (Table 1). BUSCO (v3.0.1) was used to assess the completeness of genome assembly with the insect protein set (insecta_odb9)^28^. The results showed that 94.3% of BUSCO genes were successfully detected, of which 93.2% were single-copy and 1.1% duplicates (Table 1).

### Genome annotation

**Fig. 1:**
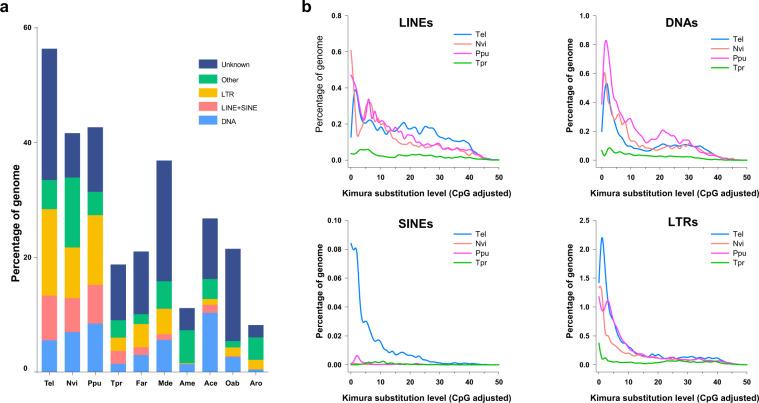
Transposable elements (TE) characteristics in the ectoparasitoid *Theocolax elegans* genome. (**a**) Comparison of TE content among 10 hymenopteran genomes. Tel, *T. elegans*; Nvi, *Nasonia vitripennis*; Ppu, *Pteromalus puparum*; Tpr, *Trichogramma pretiosum*; Far, *Fopius arisanus*; Mde, *Microplitis demolitor*; Ame, *Apis mellifera*; Ace, *Atta cephalotes*; Oab, *Orussus abietinus*; Aro, *Athalia rosae*. (**b**) Interspersed landscape of four major transposable elements (LINEs, DNAs, SINEs and LTRs). The y-axis shows the genome percent, and the x-axis shows the level of Kimura substitution (CpG adjusted) of each repeat family.

A combined *de novo* and homology-based search was applied to identify repetitive sequences in the *T. elegans* genome. The *de novo* repeat library was built with default parameters using RepeatModeler (v2.0.1)^29^. RepeatMasker (v4.0.7)^30^ was used to annotate the repetitive elements via searching against the RepBase database (v16.02)^31^ and the *de novo* repeat library. We identified 373.7 Mb of repetitive sequences, accounting for 56.4% of the assembled genome, which was the largest ratio among the 10 hymenopteran insects including two pteromalid parasitoid wasps, *Nasonia vitripennis* (41.66%) and *Pteromalus puparum* (42.67%) (Fig. 1a). Specifically, four classes of transposable elements (TEs) including long terminal repeats (LTRs), long interspersed nuclear elements (LINEs), DNA elements (DNAs) and short interspersed nuclear elements (SINEs), comprise 15.08%, 7.28%, 5.5%, and 0.53% of the *T. elegans* genome respectively (Table 2). TE landscapes based on Kimura distance values of four closely-related parasitoid wasps were calculated using RepeatMasker built-in scripts (calcDivergenceFromAlign.pl). The results indicated a recent transposition burst of LTRs, LINEs and DNAs in the three pteromalid parasitoids *T. elegans, N. vitripennis* and *P. puparum* (Fig. 1b). Moreover, a recent burst of SINEs was observed in *T. elegans*.

**Table 2:** Annotation of repeat elements in the *Theocolax elegans* genome.

Type	Number of elements	Length (bp)	Percent (%)
LTRs	141,277	99,916,631	15.08
LINEs	69,606	48,240,579	7.28
DNAs	96,901	36,431,139	5.5
SINEs	12,835	3,485,710	0.53
Rolling-circles	12,942	14,615,377	2.21
Small RNA	4,068	987,103	0.15
Satellites	8,261	3,735,317	0.56
Simple repeats	263,644	12,351,189	1.86
Low complexity	40,751	2,140,432	0.32
Unclassified	470,732	151,820,391	22.91
Total repeats	1,121,017	373,723,868	56.4

After masking repeat sequences, protein homologue searching, transcriptome sequencing and *de novo* prediction were integrated to predict protein-coding genes using the Optimized Maker-Based Insect Genome Annotation (OMIGA) pipeline^32^. For homology searching, all invertebrate protein sequences downloaded from the National Center for Biotechnology Information (NCBI) RefSeq database were aligned to the *T. elegans* genome using exonerate (v2.2.0)^33^. For transcriptome-based prediction, clean reads filtered by Trimmomatic (v0.36)^34^ were mapped to the genome assembly using HISAT2 (v2.1)^35^ and assembled into transcripts using StringTie (v1.3.4c)^36^. For *de novo* prediction, three prediction programs including Augustus (v3.1)^37^, SNAP (v2006-07-28)^38^ and GeneMark-ET (v4.21)^39^ were used. All gene evidences identified from the above three approaches were combined by MAKER (v2.31)^40^ into a weighted and nonredundant consensus of gene structures with default parameters. The predicted protein-coding genes were functionally annotated by searching against the Swiss-Prot and NCBI Nr databases using blastp (v2.8.1). Conserved domains of proteins were analysed against the Pfam (v32.0) database with HMMER (v3.3.2)^41^. The genes were mapped to Kyoto Encyclopedia of Genes and Genomes (KEGG) pathways using BlastKOALA (https://www.kegg.jp/blastkoala/)^42^, and Gene Ontology (GO) annotation was performed using Blast2GO (v5.2)^43^. Ultimately, we predicted 23,212 protein-coding genes in the genome of *T. elegans*, 20,986 (90.4%) of which were successfully annotated in at least one database (Table 3).

**Table 3:** Functional annotation of *Theocolax elegans* proteins.

Type	Number	Percent
Swiss-Prot	11,425	49.2
Nr	20,670	89
KEGG	5,532	23.8
GO	11,060	47.6
pfamA	12,293	53
Total annotated	20,986	90.4

### Orthologue and phylogenetic analyses

**Fig. 2:**
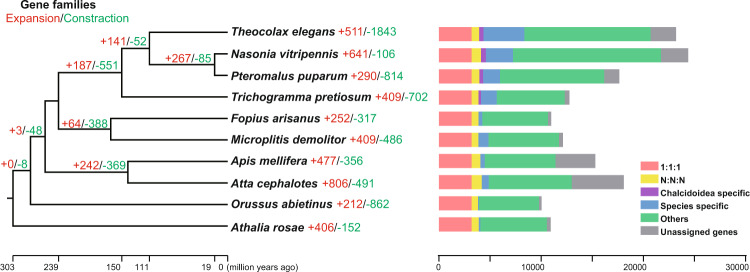
Phylogenetic and comparative genomic analyses of the ectoparasitoid *Theocolax elegans*. To the left is the maximum likelihood phylogenetic tree built from 3,199 concatenated single-copy orthologous groups from *T. elegans* and other nine hymenopterans using IQ-TREE. The basal hymenopteran *Athalia rosae* was used as an outgroup. All nodes received 100% bootstrap support. Numbers of expanded (red) and contracted (green) gene families are shown on the branches. To the right is the total gene counts of different types of orthologous groups in each genome. “1:1:1” indicates universal single-copy genes present in all species; “N:N:N” indicates other universal genes; “Chalcidoidea specific” indicates common unique genes in the four Chalcidoidea species; “Species specific” represents species specific genes with more than one copies in the genome; “Unassigned genes” indicates species-specific genes with only one copy in the genome; “Others” indicates remaining genes.

Protein sequences of 10 hymenopteran species were used for comparative genomics and phylogenomic analyses: *T. elegans* (this study), *N. vitripennis* (OGS2^44^), *P. puparum*^45^, *Trichogramma pretiosum* (RefSeq assembly accession: GCF_000599845.2), *Fopius arisanus* (GCF_000806365.1), *Microplitis demolitor* (GCF_000572035.2), *Apis mellifera* (GCF_003254395.2), *Atta cephalotes* (GCF_000143395.1), *Orussus abietinus* (GCF_000612105.2) and *Athalia rosae* (GCF_000344095.2). The longest transcript of each gene was retained for orthologue and phylogenetic analyses. OrthoFinder (v2.5.1)^46^ with default settings was used to identify orthologous and paralogous genes. A total of 3,199 single-copy orthogroups were identified and extracted for phylogenetic analysis (Fig. 2). All protein sequences were aligned with MAFFT (v7.123b)^47^ and trimmed by trimAl (v1.4.rev22)^48^. The sequences were concatenated into a supergene sequence and used for phylogenetic analysis. A phylogenetic tree was constructed by maximum likelihood (ML) method using IQ-TREE (v2.1.2)^49^, with 1000 ultrafast bootstrap replicates. The best model (JTT + F + R6) was determined by ModelFinder^50^ and the basal hymenopteran herbivore *A. rosae* was used as an outgroup^51^. Phylogenetic inference indicated the phylogenetic location of *T. elegans* in Pteromalidae along with two Pteromalinae species (*N. vitripennis* and *P. puparum*). The four chalcidoids (*T. elegans*, *N. vitripennis*, *P. puparum* and *T. pretiosum*) cluster together, with two braconids (*F. arisanus* and *M. demolitor*) as a sister group. The phylogenetic arrangement was consistent with previous studies using inference based on transcriptomic data^51,52^. Divergence times were estimated using the MCMCTree program in the PAML package (v4.9)^53^ based on protein sequences. Three calibration time points were used for calibration purposes: Chalcidoidea, 105–159 million years ago (mya), Apocrita, 203–276 mya and Orussoidea + Apocrita, 211–289 mya^45,51^. The results suggest that *T. elegans* diverged from the lineage leading to subfamily Pteromalinae (*N. vitripennis* and *P. puparum*) approximately 110.5 mya (Fig. 2). Cafe (v5)^54^ software was used to analyse gene family evolution in *T. elegans* with default parameters, and gene families inferred from OrthoFinder and estimated divergence times were used as inputs. The results suggested 511 expanded and 1,843 contracted gene families in *T. elegans* (Fig. 2). Among them, 130 and 34 gene families experienced significant expansion and contraction events (*P* < 1E-5), respectively (see table deposited at Figshare^55^).

### Gene evolution analyses

To detect genes that might be related to adaptive evolution in *T. elegans*, fast-evolving genes (FEGs) and positively selected genes (PSGs) were inferred by ratio of non-synonymous to synonymous substitutions (dN/dS, *ω*) analysis of each single-copy gene, using the branch model and branch-site model by codeml in PAML (v4.9e)^53^, respectively. Multiple protein sequence alignments were converted to the corresponding coding sequence (CDS) alignments using ParaAT (v2.0)^56^. FEGs were identified by comparing the null model (model = 0) with the alternative model (model = 2). The likelihood ratio test (LRT) was used to discriminate significance between the two models, and significance was further adjusted by the FDR method. Genes with higher *ω* values in the branch of *T. elegans* than in the background branches and FDR-adjusted *P* values less than 0.05 were considered FEGs of *T. elegans*. Additionally, PSGs at the single-codon level were identified by comparing null Model A (NSsites = 2, model = 2, fix_omega = 1) with Model A (NSsites = 2, model = 2, fix_omega = 0). Probabilities of amino acid positions with *ω* > 1 were estimated by the Bayes empirical Bayes (BEB) test implemented in PAML. Genes with positive selection sites and FDR-adjusted *P* values less than 0.05 were identified as PSGs. In total, we identified 248 FEGs (see table deposited at Figshare^57^) and 365 PSGs (see table deposited at Figshare^58^) in *T. elegans*, with 57 genes overlapping.

### Annotation and phylogenetic analysis of olfactory receptor genes

**Fig. 3:**
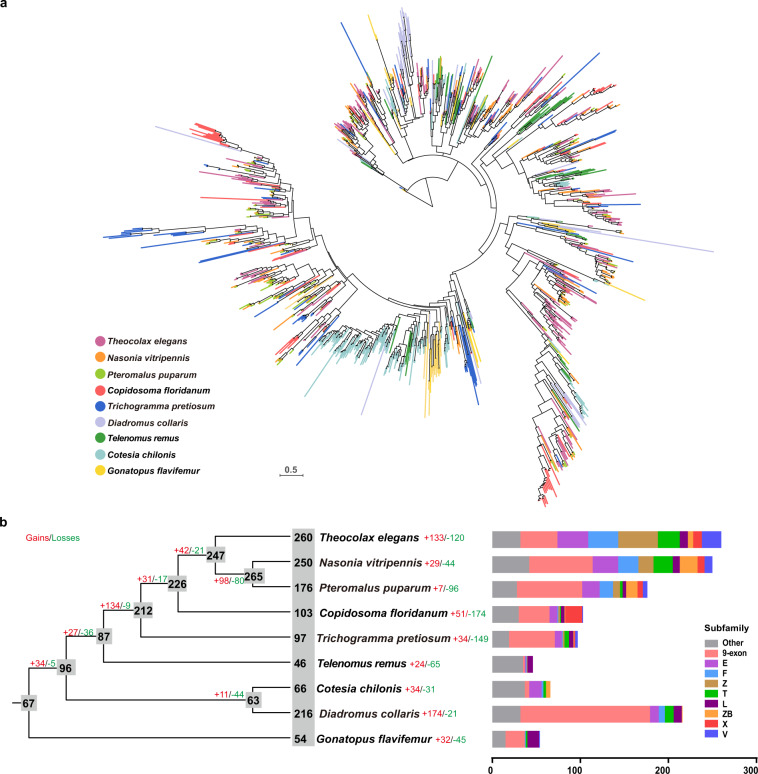
Phylogenetic analysis of olfactory receptor (OR) proteins of nine parasitoid wasps. (**a**) Maximum likelihood OR protein tree with branches coloured by nine parasitoid wasp species. Scale bar represents 0.5 mean substitutions per site. (**b**) To the left is a cladogram of nine parasitoid wasps showing estimated number of OR gene gain and loss events along branches and estimated size of ancestral and extant species OR repertoires highlighted in grey. To the right is a bar chart showing the number of each OR subfamilies.

To compare the olfactory receptor (OR) repertoire among parasitoid wasps, we annotated OR genes of *T. elegans* and eight other parasitoid wasps with high-quality genome assemblies, including *N. vitripennis*, *P. puparum*, *Copidosoma floridanum* (GCF_000648655.2), *T. pretiosum*, *Telenomus remus* (GCA_020615435.1), *Cotesia chilonis* (GCA_018835575.1), *Diadromus collaris* (GCA_009394715.1) and *Gonatopus flavifemur* (GCA_018340375.1), with the InsectOR pipeline (https://github.com/sdk15/insectOR)^59^. Well-annotated OR protein sequences from *N. vitripennis*, *A. mellifera*, *M. mediator*, *Megachile rotundata*, and *Bombus impatiens* were used as queries to search against the nine parasitoid wasp genomes. Predicted OR proteins with lengths greater than or equal to 300 amino acids and with the 7tm_6 domain predicted by InterProScan (v5.48-83.0)^60^ were defined as intact OR genes and used for further analyses^61^. All predicted protein sequences were aligned using MAFFT (v7.123b)^47^ and then trimmed by trimAl (v1.4.rev22)^48^. Phylogenetic analysis of OR proteins was performed using ML methods with the JTT + F + G4 model determined by ModelFinder^50^ in IQ-TREE (v2.1.2)^43^. Statistical support for the phylogenetic tree was assessed by ultrafast bootstrap analysis using 1000 replicates. The clade for odourant receptor coreceptor (Orco) proteins was applied as the outgroup for the phylogenetic tree. Overall, 1,268 intact OR genes were identified in nine parasitoid wasps, and extensive variation in the size of OR repertoires was observed, ranging from 46 in *Telenomus remus* to 260 in *T. elegans* (Fig. 3a,b). Gene gain and loss events were predicted using NOTUNG (v2.9.1.5)^62^ and mapped onto a species cladogram of nine parasitoid wasps inferred by former studies^51,52^. The results indicated that 133 and 120 OR gene gain and loss events occurred in *T. elegans* (Fig. 3b). OR genes were then further classified into 19 different subfamilies based on statistical support (bootstrap values higher than 70%) and subfamily definition of *N. vitripennis* OR genes in previous studies^61,63^. Among the nine parasitoid wasps, *T. elegans* has the most abundant OR genes of six subfamilies including Z (45 OR genes), E (35), F (34), T (25), V (22) and U (6), which comprise 64.2% of the OR repertoire (Fig. 3b).

### Identification of venom proteins

Venom proteins of *T. elegans* were identified using a proteo-transcriptomic approach. Approximately 100 venom glands from 3–5 d-old female parasitoids were collected separately with three replicates. RNA-Seq libraries were prepared and sequenced as mentioned above. Gene expression levels represented by transcripts per kilobase million (TPM) were estimated using RSEM (v1.3.3)^64^. Proteomic analysis was performed as described previously, with some modifications^65,66^. In brief, approximately 200 venom reservoirs were centrifuged at 12,000 × g for 10 min, and the supernatant was collected and digested with trypsin. The peptides were loaded onto a Thermo Scientific EASY Column (2 cm*100 μm, 5 μm-C18) and then separated on a Thermo Scientific EASY column (75 μm*100 mm, 3 μm-C18). Buffer A was water with 0.1% formic acid; buffer B was 84% acetonitrile with 0.1% formic acid. The buffer B gradient was as follows: 0–110 min, from 0% to 55%; 110–115 min, from 55% to 100%; 115–120 min, 100%. Resulting MS/MS spectra were searched against protein sequences using MaxQuant (v2.0.3.1)^67^ with the filtration criterion of FDR ≤ 0.01. To minimize false-positive, genes with reliable transcript levels (TPM ≥ 50) in the venom gland transcriptome and detected in proteomics with more than two unique peptides were defined as venom genes of *T. elegans*^68^. In total, 285 venom proteins were identified (see table deposited at Figshare^69^). These proteins were further categorized into enzymes (122), protease inhibitors (3), recognition and binding proteins (57), others (62) and unknown (41). The most abundant category was “enzymes” (42.8%), including serine proteases, lipase, and metalloproteases; the second most abundant category was “others” (21.8%), including heat shock proteins, major royal jelly proteins and yellow proteins.

## Data Records

Illumina, PacBio and Hi-C data for *T. elegans* genome sequencing are available as BioProject PRJNA868490 (SRA accessions SRR21010985^70^, SRR21010984^71^ and SRR21010982^72^, respectively). Illumina transcriptome data for male larvae, mixed-sexed larvae, male pupae, female pupae, male adults and female adults with three replicates are also available as BioProject PRJNA868490 (SRA accessions SRR21010979 - SRR21010981^73–75^, SRR21010994^76^, SRR21010995^77^, SRR21010983^78^, SRR21010976 - SRR21010978^79–81^, SRR21010975^82^, SRR21010993^83^, SRR21010992^84^, SRR21010989 - SRR21010991^85–87^, SRR21010986 - SRR21010988^88–90^, respectively). Illumina transcriptome data for venom glands with three replicates are available as BioProject PRJNA868589 (SRA accessions SRR21011763 - SRR21011765^91–93^), and mass spectrometry proteomics data are accessible via the PRIDE^94^ database under accession number of PXD037774^95^. The genome assembly^96^, gene CDS^97^ and protein^98^ data were deposited in the Figshare database. In addition, the genome assembly has been submitted to NCBI under accession number GCA_026168455.1^99^.

## Technical Validation

DNA quality and concentration were measured using pulse field gel electrophoresis (0.7%) and Qubit 3.0 (Thermo Fisher Scientific, USA), respectively. The integrity and quantity of RNA were evaluated using an Agilent 2100 Bioanalyzer (Agilent, USA). High-quality DNA and RNA were used for library preparation and sequencing.

## Supplementary information

Supplementary Information

## Data Availability

Software parameters of genome assembly: default parameters for HiC-Pro and BUSCO. Falcon: length_cutoff = 13000 length_cutoff_pr = 14000; pa_HPCdaligner_option = -v -B188 -M24 -t12 -e.75 –k18 -w8 –h280 –l2800 -s1000, ovlp_HPCdaligner_option = -v –B128 –h180 -e.96 –k17 –l2800 -s1000. Wtdbg:–tidy-reads 5000 -fo dbg -k 0 -p 19 -S 3 -E 5–rescue-low-cov-edges–aln-noskip; wtdbg-cns -c 3; kbm-1.2.8 -k 0 -p 19 -S 4 -O 0; map2dbgcns; wtdbg-cns -k 13 -c. SMRTlink:–bam–bestn 5–minMatch 18–nproc 6–minSubreadLength 1000–minAlnLength 500–minPctSimilarity 70–minPctAccuracy 70–hitPolicy randombest–randomSeed 1. LACHESIS: CLUSTER MIN RE SITES = 100; CLUSTER MAX LINK DENSITY = 2.5; CLUSTER NONINFORMATIVE RATIO = 1.4; ORDER MIN N RES IN TRUNK = 60; ORDER MIN N RES IN SHREDS = 60. Software parameters of genome annotation: default parameters for SNAP, GeneMark-ET, MAKER, Trimmomatic, HISAT2 and StringTie. exonerate:–model protein2genome –percent 50–score 100–minintron 20–maxintron 20000. Augustus:–species = nasonia–noInFrameStop = true–gff3 = on–strand = both. RepeatModeler: -engine ncbi -LTRStruct. RepeatMasker -a. blasp: -e 1e-5. HMMER: -E 1e-5. BlastKOALA: Eukaryotes for taxonomy group and family_eukaryotes for KEGG GENES database file to be searched. Software parameters of orthologue and phylogenetic analyses: default parameters for OrthoFinder, MCMCTree. MAFFT:–auto. trimAl: -automated1. IQ-TREE: -m JTT + F + R6 -B 1000 -T AUTO. Cafe: -p. Software parameters of gene evolution analyses: default parameters for codeml. ParaAT: -f paml -m mafft. Software parameters of annotation and phylogenetic analysis of olfactory receptor genes: default parameters for the InsectOR pipeline. MAFFT:–maxiterate 1000–localpair. trimAl: -automated1. IQ-TREE: -m JTT + F + G4 -B 1000 -T AUTO. Software parameters of venom protein identification: default parameters for MaxQuant. RSEM:–bowtie2. Custom scripts were provided at personal GitHub (https://github.com/xiaoshan40/scripts), including scripts to retrieve the longest protein and CDS sequences for each gene (get_longest_protein_and_cds.pl), to concatenate aligned protein sequences to a supergene sequence (concatenate_aligned_sequences.pl), to automatically perform gene evolution analyses (run_codeml.pl) and to extract results from the original branch and branchsite model output files by codeml (get_paml_branch_result.pl and get_paml_branchsite_result.pl).
