# Supplementary material for: Genome assembly of the ectoparasitoid wasp Theocolax elegans
Source: Sci Data. 2023 Mar 22;10:159. doi: 10.1038/s41597-023-02067-5 (PMC10033727; doi:10.1038/s41597-023-02067-5)
Supplement: Supplementary file 1 — Supplementary Information [file 41597_2023_2067_MOESM1_ESM.pdf]

Supplementary Information

for

Genome assembly of the ectoparasitoid wasp *Theocolax elegans*

AUTHORS: Shan Xiao, Xinhai Ye, Shuping Wang, Yi Yang, Qi Fang, Fang Wang and Gongyin Ye

Contents

1. **Supplementary Fig. S1 *K*-mer analysis of genome survey of *Theocolax elegans*.**

2. **Supplementary Fig. S2 Heatmap of genome-wide Hi-C data and overview of the genomic landscape of *Theocolax elegans*.**

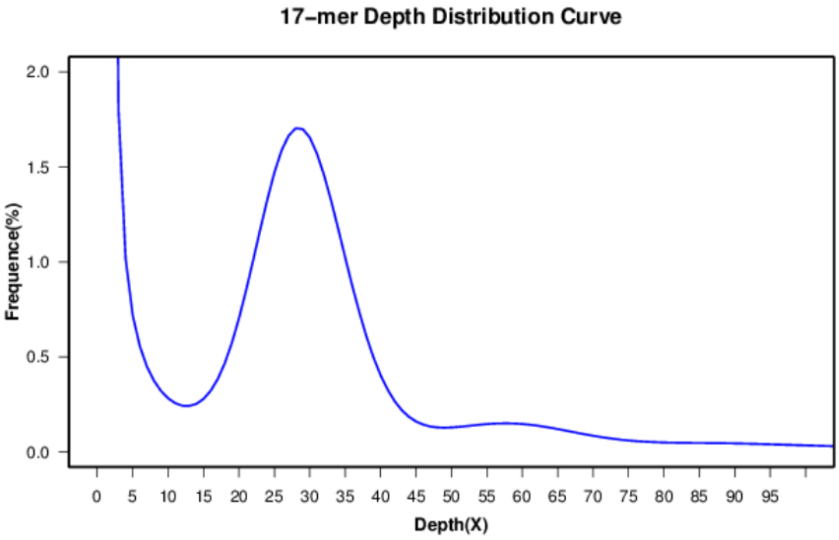

Supplementary Fig. S1 *K*-mer analysis of genome survey of *Theocolax elegans*. Genome survey sequencing data were used to count of k-mers in DNA with *K*=17.

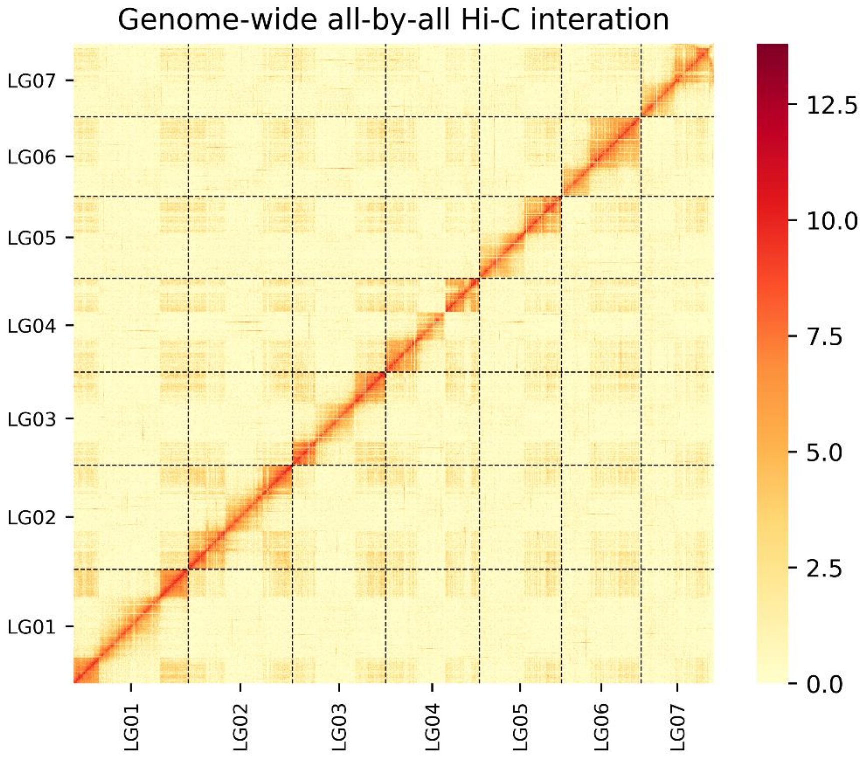

Supplementary Fig. S2 Heatmap of genome-wide Hi-C data and overview of the genomic landscape of *Theocolax elegans*.
